# “*The straw that broke the camel’s back*”: An analysis of racialized women clinicians’ experiences providing diabetes care

**DOI:** 10.1371/journal.pone.0305473

**Published:** 2024-07-11

**Authors:** Arani Sivakumar, Simrit Rana, David Rofaiel, Tehmina Ahmad, Shriya Hari, Catherine H. Yu

**Affiliations:** 1 Li Ka Shing Knowledge Institute, St. Michael’s Hospital, Toronto, Canada; 2 McMaster University, Hamilton, Ontario, Canada; 3 University of Galway, Galway, Ireland; 4 Division of Endocrinology and Metabolism, Department of Medicine, University of Toronto, Toronto, Ontario, Canada; De Montfort University, UNITED KINGDOM

## Abstract

**Introduction:**

Racialized women clinicians (RWCs) experience the brunt of unfair racial and gendered expectations, which is a direct result of their visible identity. Our study sought to understand how these experiences intersect to impact the personal and professional well-being of RWCs, and their approach to diabetes care.

**Methods:**

Data were collected from 24 RWCs working within Canadian diabetes care settings, who participated in semi-structured, one-on-one interviews conducted from April 2021 to September 2021. The data were qualitatively analyzed using thematic analysis to develop emergent themes, and interactions were explored using the socioecological model (SEM), adapted to our study context.

**Results:**

We identified three themes: (1) Discordance between self-identity and relational identity impacted how RWCs interacted with others, and how others interacted with them; (2) Tokenistic, “inclusive” organizational policies/practices and inherently racist and sexist social norms permitted acts of discrimination and led to the systematic othering and exclusion of RWCs within the workplace; and (3) Differential treatment of RWCs had both positive and negative impacts on participants’ relational, workplace and self-identity. Using the SEM, we also found that differential treatment of RWCs stems from upstream policies, structures, and social norms, percolating through different levels of the SEM, including work environments and communities, which eventually impacts one’s relational identity, as well as one’s perception of oneself.

**Conclusion:**

The differential treatment of RWCs arises predominantly from macro systems of the work environment. The burden to address these disparities must be shifted to the source (i.e., namely systems) by implementing interventions that equitably value diversity efforts, institute policies of accountability and correction of implicit biases, and prioritize an inclusive culture broadly across faculty and leadership.

## Introduction

Diabetes disproportionately impacts those with racialized backgrounds; ethnic non-European minority groups experience an increased risk of developing diabetes, compared to individuals of European background [1]. This elevated risk and prevalence of diabetes can be attributed to social, biological, clinical, and environmental factors which leads to distrust in the medical system, health provider bias, and health communication barriers [2, 3]. Structural and institutional racism are root causes perpetuating diabetes disparities amongst racialized groups; for example, inequitable access to healthcare and inadequate representation of racialized clinicians can lead to differences in treatment outcomes for racialized patients [4]. In addition, it has been shown that diabetes providers rely on observable cues, rather than clinical assessment questions, particularly age and race, to make inferences about an individual patient’s adherence to diabetes therapy. This has led to biased assessments of how racialized patients’ diabetes management is progressing. Thus, underlying differences in providers’ attitudes and communication prevail and may contribute to growing health disparities [5, 6].

Building an equitable and diverse work environment is integral to building an anti-discriminatory healthcare system [4]. Previously, it was described that patients perceived clinicians’ race as integral to cultural relatability yet viewed racialized clinicians as less professional [7]. While patients preferred female clinicians over male clinicians, this stemmed from sexist perceptions of femininity associated with “caring work”, which led patients to also exhibit greater instances of role misattribution and deprofessionalization towards female clinicians [7]. The manifestation of gender stereotypes assigned to professions has been reported in previous research; women physicians of color suggested that they are often misidentified as nurses or custodial staff by colleagues, which they perceive to be a culmination of their intersectional identity [8]. Different power dynamics exist for different subgroups of women clinicians, which influence their experiences of discrimination [8] These paradoxes compel a greater need to investigate how racialized women clinicians (RWCs) navigate their work environments; specifically, the impact of racial and gender expectations within their daily interactions.

The effects of discriminatory practices do not exist in siloes, but sweep across all domains: occupational, social, physical, and mental well-being [9]. Much work has focused on patient and physician race/ethnicity or gender and their associations with the patient experience and specific aspects of the patient-physician relationship. However, little research has understood the interaction from the health provider perspective, particularly given the added diversity in the workforce.

We sought to understand how racial and gendered expectations of patients and colleagues intersect to impact RWCs’ personal and professional wellbeing, and their approaches to caring for patients with diabetes. A secondary objective was to apply the SEM to RWCs’ experiences, so as to identify interrelated factors that can be leveraged to propose potential solutions within the context of the healthcare system.

## Methods

### Study design

This study utilized a qualitative research design, mapping our findings to SEM constructs [10], which have been adapted to the study context (Table 1). Our study was reported according to the Consolidated criteria for Reporting Qualitative research (COREQ) (S1 Table) [11].

**Table 1:** SEM construct definitions adapted to the study context.

SEM Constructs	SEM Definition	Definitions Adapted to Study Context
Identity	Who they think they are	Who they self-identify as
Relational/relationship identity	How they view themselves based on local external factors, such as friends	How they view themselves in relation to supervisors, colleagues, and patients
Work environment	How organizational norms and behaviours affect the individual	How the workplace environment affects their perceptions/feelings of inclusion
Policies/societal norms	How societal norms and policies affect the individual	How societal norms and policies affect their perceptions/feelings of inclusion

#### Use of theory: Socioecological Model (SEM) framework, intersectionality and theorizing gender and race

We employed the socioecological model (SEM), a theoretical framework which posits that an individual’s health outcomes and experiences are impacted by, and occur within, a larger, dynamic network of intrapersonal relationships, characteristics, relational factors, institutional processes, and societal norms and expectations [10]. The paper also draws on theories of intersectionality [12], which posit that experiences of neither gender nor race can be addressed *singularly*; rather, the *combined* experiential impact of race and gender affect racialized women at all levels of their professional lives. In this study, racism was conceptualized as a dynamic process that operates across the systemic, structural, and individual levels [13], as it can occur systemically through policies, practices and institutions, but also socially through interpersonal interactions. At the individual level, racism may be perceived as certain ideas about one’s racial identity. At the interpersonal level, racism is conceptualized as other people’s ideas about their racial identity. At the institutional and systemic level, racialized individuals navigate complex policies, regulations and circumstances that exacerbate disparities in resources, and opportunities. Finally, in our conceptualization of gender and sex, we make a note here that sex and gender are not the same and highlight the interplay between internal (felt) identity and externally mediated norms and expectations of gender or “sex” that people continually navigate. Thus, the utility of the SEM is that it allows for analysis across all levels of influence, interaction, and norms. For example, we can readily analyze how individuals understand *their own* experiences of race and gender; we can also readily observe how interpersonal interactions, institutional norms, and systemic/structural policies also shape individuals’ experiences of their racialized and gendered identities. Further details regarding theoretical underpinnings are included in S1 File.

### Setting and participants

We focused our attention in diabetes care, given the predominance of racialized populations in this patient group, and the consequent relevance of having a racially diverse health care workforce to serve this population [4]. Our research question centered on women, given our previous findings that demonstrated a paradox of patients preferring female clinicians yet simultaneously deprofessionalizing them [7]. We recruited from hospital- and community-settings, to ensure diversity in clinician roles and experiences. Convenience sampling was employed by reaching out to individuals involved in diabetes care from clinical, academic and research networks of research team members across Canada. Virtual snowball sampling was also conducted through social media posts to online diabetes health and research networks to expand the geographical scope of our study, so as to ensure better representation of professionals engaged in Canadian diabetes care. By this method, we recruited RWCs within academic and community settings across Canada. Inclusion criteria for our study were: (1) participants self-identified as female, (2) identified as racialized, and (3) was a clinician delivering diabetes care within Canada.

### Data collection

The study was approved by the University of Toronto Health Sciences Research Ethics Board (Protocol #40440) in February 2021. Prior to conducting interviews, a demographic questionnaire was emailed to prospective participants, elucidating sociodemographic and practice characteristics (i.e., age, race, role, number of patients seen per week, percentage of practice comprising patients with diabetes). This data was collected in order to utilize an intersectional framework when analyzing participants’ experiences and ideas of their own experiences. Gender-related data was not collected because the inclusion criteria defined study participants as “racialized women”; all participants therefore were women. Race-related data was collected to elucidate specific racialized identities of participants in order to better understand how racialized individuals from different backgrounds experienced racism. Individuals who met the inclusion criteria were invited to participate in a 45- to 60-minute individual interview and were provided with an informed consent form. Written informed consent was voluntarily obtained from participants prior to scheduling their interviews, and informed consent was verbally reassured prior to starting the interview. Data were obtained through semi-structured, individual interviews, conducted in English from April 2021 to September 2021.

Interview guides consisting of open-ended questions explored the racial and gendered aspects of one’s perceived identity, racial and gendered makeup of the workplace and workplace expectations, inclusion, and experiences in relation to one’s identity. Interview guides were pilot tested on members of the research team to assess face validity and were refined accordingly. Field notes were made during the interviewing process to not only document the various factors informing participant meaning (e.g., tone of voice, pauses) but to also promote reflexivity by outlining analytic insights and speculations. This form of reflection after each interview allowed the interviewer to assess their biases and feelings. Field notes contained immediate and later thoughts about the research process, which were then discussed during regular research team meetings. Trustworthiness of the study [14] was ensured by keeping field notes, engaging in investigator triangulation (i.e., coders independently analyzed the same qualitative data and compared findings to seek the multiple meaning) [15] and environmental triangulation (i.e., recruiting participants from different settings (academic and community) or regions of the province/country to use more than one context to study the intended focus) [16]. Interviews were conducted by three research team members, trained in qualitative interviewing (AS, SR and DR), using Zoom [17]. As this study took place during the COVID-19 pandemic, our data collection was limited to virtual interviewing as opposed to face-to-face interviewing, due to social distancing measures. Interviews were digitally audiotaped, transcribed verbatim and annotated with field notes. Participant-identifying information was replaced with de-identified details, and all participants were assigned a randomly generated code. The master linking log is stored on a secure server and only accessible to members of the research team.

### Data analysis

Each transcript was analyzed individually using constant comparison and content analysis [18]. To establish intercoder reliability and ensure analytic rigour and trustworthiness, we had a minimum of two trained coders (AS, SR, DR), with at least one member removed from the data collection to address any potential biases [19]. Coders adhered to the Framework Method [20], and maintained field notes as well as meeting minutes, which served as thematic logs and audit trails [15]. Specifically, we coded each transcript, categorized each code, and developed a coding framework, which was iteratively refined with ongoing coding [20]. As we coded transcripts, we identified and highlighted similarities and differences. The coders used the same coding framework to ensure that basic themes and concepts established from previous iterations of the framework were developed. For example, codes such as “Navigating multicultural identities” and “Appears ethnically ambiguous” would fall under the broader theme of “Identity”. Coders focused on the shared meanings from the coding framework and confirmed codes through dialogue and consensus. Any discrepancies in codes were discussed during research team meetings, whereby other members with qualitative coding experience were consulted for their expertise to resolve discrepancies. Data saturation is described as the point at which new data become redundant with previously collected information, as reiterated by Sandelowski (2008) and Grady (1998) [21] This redundancy is observed when researchers encounter repetitive comments or themes during interviews. In the context of qualitative analysis, saturation occurred when no new codes or themes emerged from the data, which then permitted the development and application of an SEM-conceptual framework (Table 1). These codes were further examined to identify connections and relationships between the multiple levels, so as to inform our discussion. NVivo version 11 [22] was used to facilitate data analyses. Researcher positionality are noted in S2 File.

## Results

### Participant characteristics

A total of 24 one-on-one interviews were conducted with RWCs working in hospitals, clinics, and community health centers. Participant demographics are listed in Table 2 by racial identity, clinician role, practice setting and province of practice.

**Table 2:** Demographic characteristics of RWC study participants.

Demographic Characteristic	Number of Participants (%)
**Racial Identity (n = 24)**	
East Asian	9 (37.5%)
South Asian	5 (20.8%)
Black	6 (25%)
Indigenous or Métis	1 (4.2%)
Mixed	3 (12.5%)
**Clinician Role (n = 24) and Participant Identifiers**	
Physician (MD1—MD9)	9 (37.5%)
Nurse Practitioner (NP1)	1 (4.2%)
Registered Nurse (RN1—RN7)	7 (29.2%)
Registered Dietitian (RD1—RD5)	5 (20.8%)
Social Worker (SW1)	1 (4.2%)
Pharmacist (PH1)	1 (4.2%)
**Practice Setting (n = 24)**	
Academic	12 (50%)
Community	12 (50%)
**Province of Practice (n = 24)**	
Ontario	19 (79.2%)
Quebec	1 (4.2%)
Alberta	3 (12.5%)
Yukon	1 (4.2%)

### Findings

#### Theme 1: Discordance between self-identity and relational identity impacted how RWCs interacted with others, and how others interacted with them

Subtheme 1a: One’s self-identity is intimately linked to, yet distinct from their relational identity

When participants were asked about their perceptions of self vis-à-vis race and gender, we identified two discrete response types: who they think they are (“self-identity”) and how they see themselves in relation to others (e.g., colleagues, patients, family and friends) (“relational identity”). For example, one participant labelled herself as “Canadian, with maybe a bit of a Chinese background” (Table 3, Quote 1). She further elaborated by considering her identity relative to two distinct cultures (Canadian, Chinese); who she was (i.e., personality traits, behaviour) was in the eye of the beholder (Table 3, Quote 2).

**Table 3:** Illustrative quotes from RWC participants.

Descriptor	Quote
1) Canadian identity mixed with Chinese background	*“I think tricky question ’cause like in terms of race like really just Canadian*, *’cause like I feel like I was born in Canada*, *I grew up in Canada so I’m mostly Canadian*, *I’m aware that like for ethnicity*, *I come from like a Chinese background so there is a component of that*, *maybe like a bit of a Chinese Canadian identity*, *like I’m aware of that I’m a little bit more*, *not as attached to Chinese culture*. *I don’t really identify as Chinese like I don’t speak Chinese I don’t understand Chinese*. *Yeah*, *so concerning gender*, *female Canadian*, *with maybe a bit of a Chinese background*.*”* [MD2]
2) Defining identity in relation to two distinct cultures	*“I think that’s consistent*, *kind of*, *with growing up between 2 cultures ’cause I’m aware that with Canadian culture where you are expected to ask a lot of questions and kind of put yourself out there and kind of bring attention to yourself*, *and then there’s sort of Chinese culture where that’s very much*, *you know*, *not how you behave*, *and first it and kind of like more subtle or a little bit different*, *and so sometimes I have elements in both of those and that personality and I think any people can sometimes*, *you know like there’s different… like I guess if they see the way I look and they see the way I behave*, *you can see that I come from different cultures*. *I would also say that if you’re purely Chinese you would see that I’m not ‘Chinese’*, *just from the way that I speak*, *in the way that I behave that I’m definitely not like someone who was born in China*.*”* [MD2]
3) Relationships and interactions shaped by strong matriarchal influences and culture	*“First Nations people were traditionally a matriarchal society and my mother was very much raised by her grandmothers and her mother so she raised me to be very strong in myself as a woman and to pursue education*. *It was her that pushed me all the way so I think it has given me a lot of confidence*, *because I wasn’t raised in a traditional Western gender role that I should be pretty and shut the fuck up so yeah*, *she’s taught me to speak my mind*. *She always spoke hers*, *she didn’t say much but she did*.*”* [RD5]
4) Perception as personable leads to increased time spent with patients	*“With patients*, *I do get to know them and ask a few questions outside of their medical issue just to get to know them a little bit more and know their kind of situation*.*”* [RN3]
5) East Asian RWCs read as younger	*“I’m female*, *I’m small*. *I guess*, *sometimes people say that Chinese people age fairly well*, *so they look younger*.*”* [MD1]
6) Confidence in RWCs’ clinical expertise is undermined until proven.	*“It was very much earlier in my career*, *I was going to give a talk to a group of cardiologists*, *and I walked in and the chair-person sort of looked at me […] he was just not a nice man*, *and was sort of patronizing and condescending*. *And then I did my talk*, *and then after that*, *he was all friendly and ‘that was great*, *thank you very much’*. *So*, *there was an assumption that he made when I walked in the room that I wasn’t going to be any good*. *Now*, *again*, *was that race*, *was that gender*, *was that age*? *I suspect it’s all of the above*, *lumped in there*. *Plus*, *there were endocrinologists and cardiologists […]*. *But if I really think about it*, *let’s say I had been a middle-aged white male who walked in*, *in a suit*. *Would he have treated that person differently*? *And I think the answer is yes”* [MD3]
7) RWCs’ workload is different from non-RWCs	*“I identify myself also as Tamil so when I go in there*, *[…] a lot of people would be like*, *“Oh*, *you’re Indian so you must know how to make this”*. *And you’re like*, *“No*, *I don’t”*, *like you can’t just assume that just because of my skin tone*. *So yeah*, *I’ve had a couple of coworkers say that*. *Sometimes a lot of tasks they do get*, *my manager would ask me*, *“Hey*, *can you do this*? *I know you usually go above and beyond*.*” So*, *I think it depends on also what your manager sees about your work ethic*. *So those two things are what I’ve noticed presently*, *because sometimes I do realize my workload compared to someone else is different*, *like another dietitian or diabetes educator and that gets picked up by other people such as your manager and your peers”* [RD2]
8) The expectation to take on a greater workload.	*“I kind of feel like I have to take on more*, *do a little bit more*, *probably be about maybe 60% of taking on a larger workload or working longer hours”* [RD4]
9) Patient-originating racism: Patients routinely question credentials of RWCs.	*“They said ‘Why did you do this and not that*?*’ Then I have to explain where I read that you can do this and not that one*. *But you will notice that they don’t do that with other colleagues but I have noticed that people from different*, *like minority backgrounds*, *like if you’re not Caucasian*, *you are in trouble”* [RN3]
	*“I think you’ll have people who make comments about how good your English is so*, *I think that when those things come up*, *and I get the sense that people are challenging my competence*, *or are concerned about my level of skill or background”* [MD8]
10) Patient-originating racism: racist commentary by patients	*“There was an incident where we had a client come in*, *and he was a white man*, *who was very adamant that single white middle-aged men were like the most forgotten people in Toronto*. *And so he would say very racist things like*, *you know*, *we need to defund Black Lives Matters […] When we started this conversation*, *things like entitlement that often comes with my white clients and it doesn’t matter if they’re marginalized or not so that it doesn’t matter what income bracket or socioeconomic status they come from*, *there is the expectation that they’re entitled to my time*.*”* [RD1]
	*“It’s only more awkward when they don’t realize that I’m First Nations and they go on about the stereotypes of how stupid First Nations people are and then they start looking around at everybody’s chins kind of dropping to the ground*, *and then they’re looking at me […] awkward things like that*. *[…] And then they go*, *“Oh is something going on here*? *I didn’t mean it like that*!*”* [RD5]
11) Overt racism stemming from current political context.	*“I think in the beginning of the pandemic*, *I did encounter […] one instance*, *in which a colleague identified my particular race as the cause of the pandemic*.*”* [RN4]
12) Overt racism toward Black RWCs from upper management.	*“I feel that she directly targeted people that were African Canadian*, *and always tried to be confrontational or be unsupportive or “get them in trouble”*. *So definitely I thought that was racism*.*”* [RN7]
13) RWCs are are invisibilized	*“I have a co-worker that will come to work*. *[…] We have this quick weekly meeting where we go for like an hour once a week and for two years*, *this person will come in the meeting and look at me every day and say “So who are you again*?*” Two years that I was working in this place*. *So one day one of the people couldn’t take it anymore*, *somebody actually spoke up for me because this person was in a senior position so I couldn’t really just snap at his face because as an African*, *we are taught that if somebody is born one day before you*, *you have to respect them regardless*. *So I couldn’t snap but somebody one day came to my defense and said*, *“Oh*, *let me tell you who she is*.*” […] At the beginning*, *it makes sense*, *right*, *because you’re new but then after a week*, *a month*, *two months*, *a year*, *he would still say that*, *every time I came*. *[…] After two years*, *you don’t know who I am so what am I supposed to do*? *[…] When people leave you on the side*, *you just be on the side and do what you could from that side*. *So if people are acting like this*, *how would you feel included*?*”* [RN3]
14) Institutional tokenization of RWCs not consulted for content, only included for tokenized “representation”	*“They’re not familiar with people like me and they don’t know how to act or interact with people like me because at their table there is a significant lack of diversity at the senior level*. *And so*, *their collaborations or integrations with people like me is on the politician level where they don’t have real conversations*, *it’s more so disingenuous appreciation for what you do*, *but not understanding who you are as a person or individual*.*”* [RN6]
15) RWCs reduced to visible-identity	*“Inclusion committees are a way to silence those wanting real change*. *Well definitely*, *I think it’s that inclusivity*, *trying to make it look like we’re okay*. *‘We do all of these other things but just to shut them up; let’s give them this one*. *That’ll shut them up for a little bit*. *Look they can’t say this because we have one here’”* [RN7]
16) Work environments are tailored to prevent/discourage RWCs from speaking up.	*When superiors are white*, *there’s a lot less sort of openness*. *So specifically*, *one that I remember*, *a superior was a white lady who has basically maintained a very curated environment where a lot of me and my racialized colleagues are often not able to speak up about our experiences*. *[…] There’s often the suggestion that our experiences are always disproportionate or we’re making a mountain out of a molehill*, *especially because the experiences of racialized women*, *often it’s not empirical*, *it’s not something you can touch; you can say but you can’t name*. *We tried speaking up a few times but there was always that threat of my colleague being fired*. *At the end of the day*, *to her*, *it’s her livelihood*. *It’s not worth fighting for*.*”* [RD4]
17) RWCs are expected to tolerate instances of overt discrimination.	*“I’ve had multiple conversations with multiple staff who have had racial slurs like the N-word*, *like several Islamophobic terms railed at them for things such as denying a person the keys to the washroom because it’s a staff washroom not client washroom*, *that was the reaction that they got*. *[…] A manager would come down and calm down the client*, *open up the door*, *let them use the washroom and walk them out of the facility*, *and come back to the staff person and say*, *‘Um*, *you know*, *this person has severe mental health and we need to be compassionate*, *and are like*, *so sorry*, *you had to deal with that’*. *But the unspoken thing is really*, *like*, *‘You need to suck it up’*. *I think it’s definitely a culture in the organization to feel like*, *‘Oh*, *it’s a harmless thing that you know*, *it’s not coming from somewhere real but we’ll tolerate it’”* [RD1]
18) DEI initiatives are tokenistic and not led by RWCs.	***“****Even seeing what work is being done*, *it’s kind of like a checkbox and it’s funny that when you’re doing anti-racism work*, *the recommendation is that those who have experience should not be the ones that are leading the work*. *I understand the fundamental reason behind that because you want those who are the oppressors to recognize what they’re doing and to address the systemic racism that exists*. *The challenge here is that when you put individuals who are uncomfortable with the topic and the experience*, *the strategies and action plans that are going to be employed are not necessarily helping those who have experience but making the individuals who are leading*, *feel better*. *So*, *they tend to select strategies and action plans that make them feel better*, *or are manageable and actionable*, *but in actuality*, *you’re no further ahead […] I was thinking here’s this organization putting out all these lovely fluffy emails about identifying racial disparities and supporting our racialized staff members and you know*, *creating this counsel*, *but I’m thinking you have these types of people working here*? *There are so many grievances*, *and everybody looks the same*. *So here you are sending out these emails*, *but do you truly uphold these standards*? *Do you really believe in these values*? *But is this because what’s going on in the media now*, *it’s now more prevalent*, *that now we have to take this response*? *Do you truly care*? *Do you truly get it*? *[…] Like hasn’t been going on for hundreds of years*? *[…] It’s just talk*. *I don’t see that represented in the core values by the staff members that are here*.*”* [RD6]
19) Expectation by upper management to tolerate racist expressions from patients	*“I think my manager just expects me to tolerate things even if they’re damaging to me*, *like in that situation that I described earlier on like*, *that I’ll suck it up and do it and I think it’s subconscious*, *I don’t know if she’s doing it with full knowledge of like how it might impact me*.*”* [RD1]
20) Onus unjustly placed to act as a cultural/anti-racism ambassador in workplace	*“To me it’s an injustice and it’s unfair to be like… again there’s 3 individuals*, *so there’s a very small group of individuals who at one time in life have felt that*. *To stand up and fight against that is exhausting*, *like if I had more colleagues who had similar*, *not same but similar*, *it would be less of a daunting task to change the environment*.*”* [RD3]
21) Perceived race-based discordance in the resources provided to new employees	*“Because when there are newer staff that identify as Caucasian*, *I feel like the support*, *and their education and their training is set up much more differently*, *that only supports them to be successful*. *And you’re kind of just thrown into the fire and have these high levels of expectations*, *not necessarily given that same level of cushioning and support*.*”* [RN7]
22) Minimal to no accommodations provided for career development	*“I was interested in pursuing my Masters and other personal interests that I have*, *like the support was not there*. *‘Oh we don’t think*, *we’ll see about your schedule’… even just asking for reference*, *I was told that absolutely*. *I had a meeting*, *and I was strongly encouraged; ‘This will open up so many opportunities*.*’ I submitted my application for my masters and the reference was not submitted which I could see*. *I talked to my colleague*, *‘How long do I wait before questioning and asking my superior if she can remember to complete it on my behalf’ because it was such an encouraging response when I initially discussed that I had these plans*. *And basically she said to me; ‘Ph find someone else I don’t want to ruin your chances*.*’ After my application was already submitted*. *I had to then go to a previous manager that I worked with for a much longer time*, *find her*, *which she had moved on from the organization*, *I had to find her and then beg the university to change my referee because she just completely was going to sabotage my success*. *Whereas*, *the new [white] grad*, *she was completely supported*, *even was able to keep her full-time status*, *do her curriculums*, *do her placements*. *Even though it often times was overlapping with her full-time role*, *her complete schedule was accommodated*. *I didn’t get that*.*”* [RD7]
23) Solely racialized working groups contribute to the stagnation of transformational change	*“I also think that in terms of staff*, *I’ve mostly talked to racialized staff so I think one of the things that we realized early on in this working group is that there isn’t*, *everyone who’s on the working group is racialized*. *And so*, *there aren’t enough allies within our organization that are engaged in this process or see themselves or see their role within it*.*”* [RD1]
24) White allies identified as facilitators to RWCs’ career advancement.	*“There was a higher seniority nurse practitioner who actually did a rotation here*, *so she was more experienced*, *she had her CDE and even with all that background*, *she was not supported for that position*. *There was like a big uproar and pushback*, *and ultimately the reason why she got that position was the department head stepped in and said*, *‘Absolutely not*, *she’s qualified for this position*, *she has the seniority*, *she’s deserving of this position and advocated for her*.*’”* [RN7]
25) Whiteness linked to authority and thus entitlement	*“A comment that always stuck out to me was ‘Do you know who I am*? *I am so and so’ and I often don’t know who these people are so I would say no*. *The patients would then you know make it known that they are of a certain stature or status in the hospital and hold certain privileges just to get out of having a conversation with me which is very interesting*. *[…] They were all white males*, *interestingly enough*.*”* [RD4]
26) Assertiveness exhibited by RWCs may be perceived as aggression.	*“I work in an all-woman female team*, *I often think that we would benefit from male energy because they often feel like men are normalized to have more decisive*, *more upfront*, *more direct conversation and dialogue*. *And I think it’s in a woman*, *it’s perceived as aggression but I think there’s lots of value in that type of approach*, *especially when you’re having cyclical conversations and people can’t make decisions and no one’s taking a leadership role and all those things that often happen*, *I think*. *And when I am direct*, *I often stress about how I am being perceived*.*”* [RD1]

*Relational identity* was shaped by community (not only by upbringing but also the prevailing worldview); for example, one Metis participant’s relationships and interactions with the world were formed by a confluence of a strong female role model (i.e., her mother) within a matriarchal culture (First Nations) (Table 3, Quote 3).

Participants shared that they struggled with this disconnect between self-identity *(“what I experienced in my own home”)* and relational identity *(“who I see out in the community/how I am seen by people in different communities I am in”)*. In particular, this was a recurrent theme for first generation Canadians:

*“It’s an ongoing identity […] struggle*, *[…] like “Are you Canadian*? *Or are you the type of culture and race and ethnicity that you’ve been brought up with in your home*?*”*

[SW1]

This identity “struggle” did manifest into loss of a sense of inclusion, in either group:

*“Chinese individuals don’t see me as Chinese*, *so […] not only do I not necessarily feel accepted by the Canadians who I use to identify myself*, *I’m not necessarily accepted by the Asian community either because they don’t speak the language*.*”*

[RD3]

Subtheme 1b: Discordance between self-identity and relational identity framed their approach to relationships, as well as others’ approach to (and differential treatment of) them, colouring their experiences in delivering care

Participants shared that both self-identity and relational identity contributed to their approaches to clinical care. For example, some participants self-identified as being personable, and this motivated them to spend quality time with patients (Table 3, Quote 4).

Similarly, relational identity contributed to their approach to care; concordant identities appeared to enable productive interactions, whereas discordant identities required explicit management of others’ expectations. Specifically, when concordant with self-identity, we noted that relational identity related to ethnicity appeared to facilitate communication and a sense of inclusion:

*“Sometimes being a black person*, *when you speak to some patients who identify with you*, *they are more comfortable discussing with you*. *So for me*, *that’s part of being included*.*”*

[NP1]

In parallel, relational identity related to gender also facilitated rapport when concordant with self-identity:

*“It’s my gender that makes a difference because I’m running around chatting with people*. *I’m more likely to strike up a conversation and randomly talk to everybody*, *than either of my male colleagues*.*”*

[MD6]

We also identified circumstances where relational identity associated with gender was discordant with self-identity: this impacted expectations of gendered care by patients. One East Asian participant hypothesized that this discordance between self-identity and relational identity was a consequence of socialization:

*“There’s a perception of women being more empathetic and more talking about feelings and soft stuff*. *That’s how we’re taught*, *how we’re socialized*, *and I think it’s to the detriment frankly of men who are taught the opposite right*? *They are taught […] you’re not supposed to be soft; you’re not supposed to talk about feelings; you’re not supposed to show vulnerability […] That’s the perception society has and therefore I’m not surprised that patients would perceive that they should not talk to a male doctor about their problems but they’d be more comfortable speaking to a female doctor about them*.*”*

[MD3]

**Fig 1:**
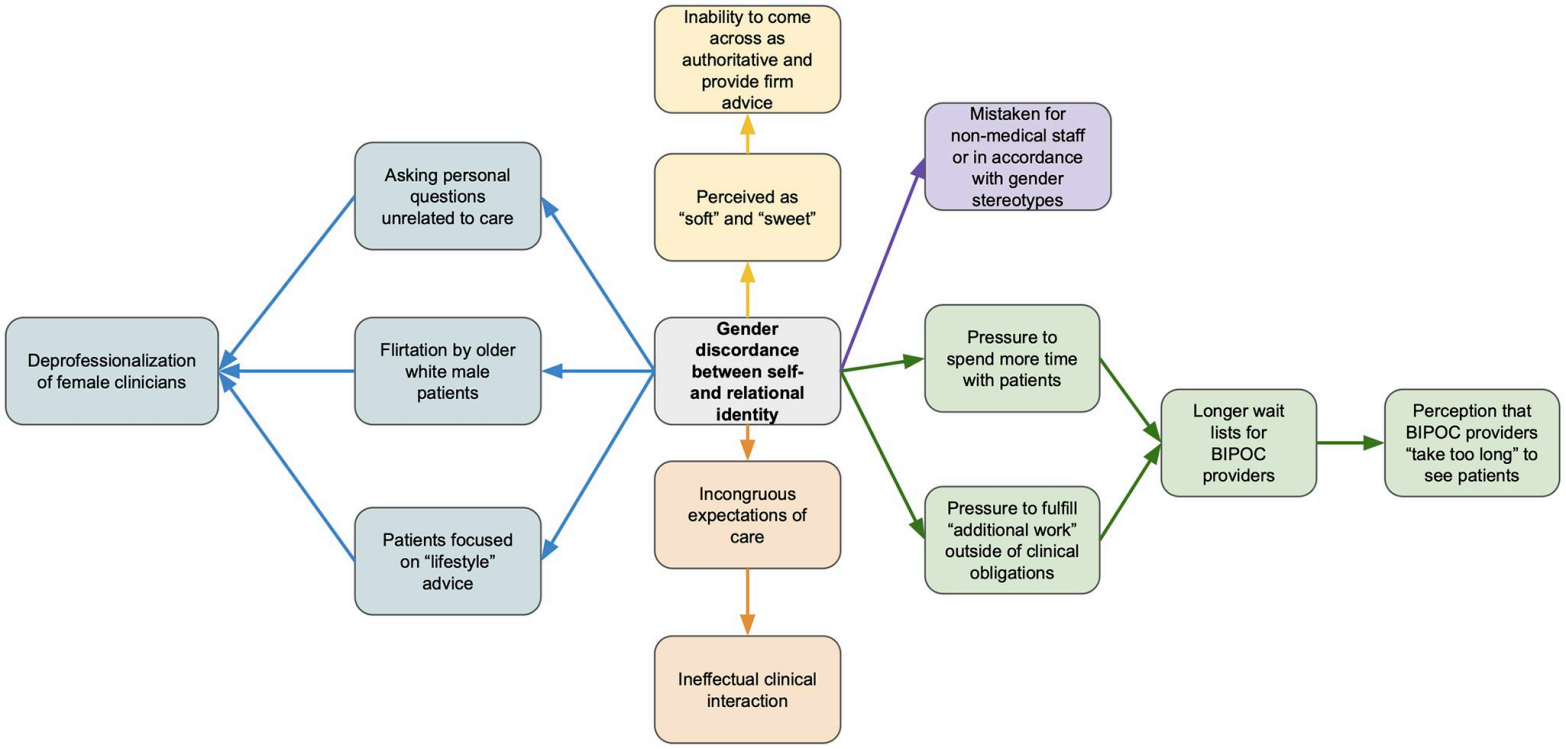
Manifestations and impacts of gender discordance between self- and relational identity.

This gender discordance between self- and relational identity was found to impact various facets of care delivery. In accordance with gender stereotypes, participants reported being mistaken for non-medical staff, were faced with incongruous expectations of care, were deprofessionalized, felt pressured to surpass clinical obligations, and experienced a disproportionate number of referrals (Fig 1). These racist and sexist norms intersect with perceptions of younger RWCs because youth was viewed as less professional, less experienced, and less authoritative” (Table 3, Quote 5).

This “additional work” and deprofessionalization creates a profound paradox of simultaneous low and high expectations (Table 3, Quote 6): low expectations exist because of the consistent othering and marginalization of RWCs as inexperienced, less educated, and incompetent, while concurrently, RWCs are also expected to meet *higher* expectations of greater relatability with BIPOC patients, more diverse skill sets and heavier workload (Table 3, Quote 7,8) (i.e., sitting on committees, more thorough conversations with patients and “taking time to listen”). Thus, discordance between self-identity and relational identity resulted in incongruent expectations with patients, colleagues, and supervisors; taxing their time, energy, and effectiveness. Additionally, these incongruent expectations can create challenges for RWCs who attempt to create healthy boundaries regarding the work they are engaged in.

Subtheme 1c: This differential treatment from patients and colleagues, as a result of the discordant identities, often manifested as overt racism directed towards the participants

Participants reported experiencing overt racism from patients: patients made requests for English-speaking clinicians based on RWCs’ names, accents, racial identities, or mannerisms (Table 3, Quote 9). They shared that patients routinely questioned their credentials, education, and expertise, asking “Why did you do this and not that”, whereas their white male counterparts were “never questioned.” To compound this, participants divulged that they were expected to listen to racist commentary from patients:

*“It’s awkward when they don’t realize that I’m First Nations and they go on about the stereotypes of how stupid First Nations people are*. *Then they start looking around at everybody’s chins […] dropping to the ground*.*”*

[RD5]

Canadian-accented English was seen as *“more professional*, *more white*, *more normal*, *more educated”* (Table 3, Quote 10). The same East Asian participant thoughtfully identified that:

*“There’s an implicit racism*. *It’s just the fact that our society is white-gaze attuned*.*”* [MD8]

Participants also experienced racism from colleagues, which ranged from subtle slights associating incompetence with “other” cultural backgrounds, like: *“Okay*, *because you’re from this culture*, *it doesn’t mean that things are being done like that*, *it should be done this way*. *This is how we do it here*.*”* [NP1]; *t*o overtly racist comments such as *“they accepted you because you are Asian into this program”* or *“they picked you for that program because you are coloured*.*”*

Overt examples of racism ranged from the case of “a colleague [who] identified [her] particular race as the cause of the pandemic” (Table 3, Quote 11), to experiences with a former manager who “directly targeted people that were Black, and always tried to be confrontational or be unsupportive or get them in trouble” (Table 3, Quote 12), to total erasure of a participant’s presence, skills, and contributions due to invisibilization by a white colleague who persistently denied her presence for over two years (Table 3, Quote 13), to egregious comments condoning genocide by way of displacement:

“*I had one surgeon*, *when there was an Inuit girl who was pregnant*, *who was sniffing or using some sort of substance that was very harmful to the fetus*. *He wanted to know what my thoughts on that were*, *because he was pretty sure that First Nations people were becoming an inferior race*, *and that his solution to all of the alcoholism and revolving door stuff that was going on was because you know*, *all First Nations people are artistic*, *that we just get them on an island*, *we get them some art supplies*, *but we have to make sure we cut down all the trees so they can’t build rafts and get away*. *And we’ll just send them some art supplies and food and we’ll have it all dealt with*”

[RD5]

#### Theme 2: Tokenistic, “inclusive” organizational policies/practices and inherently racist and sexist social norms permitted acts of discrimination and led to the othering of RWCs within the workplace

Subtheme 2a: Healthcare organizations decide what is or is not racist without meaningful BIPOC input and consultation

Workplace cultures perpetuate differential treatment of RWCs via racism and misogyny through tokenized “opportunities”, whereby RWCs’ comments, questions, or analyses at meetings are not considered as seriously as their white, male counterparts. In this manner, RWCs may serve on committees, Diversity, Equity and Inclusion (DEI) boards, or in other capacities but learn that their presence is reduced to merely their visible identity as understood through stereotype, and that they are not often consulted for content, strategic directions, planning, or executive decision-making (Table 3, Quotes 14).

This “reduction-to-visible-identity” is often disguised at the workplace level through inclusionary language that conceals the tokenistic role into which they are slotted (Table 3, Quote 15). For example, a Black registered nurse recounted that she was:

“*Nominated for an inclusion award*, *and one of the comments that two physicians said is that ‘she runs the best holiday parties and the best entertainer*.*’ So*, *I’m thinking*, *what about my operational skills*? *What about my knowledge*? *What about my ability to run a diverse team*? *So again*, *following the stereotypical aspect is that what people see first is not the knowledge and the expertise*”

[RN6]

Organizations may also have their own norms that reflect wider societal norms as to what may “count” as racism or effective responses to racism. RWCs noted that these norms of what is considered “racism” often limited their ability to express their experience and effectively name moments of discrimination, resulting in a “curated” version of reality (Table 3, Quote 16). Relatedly, BIPOC staff felt unsupported by their organizations, stating that they did little to mitigate the racism directed at them by patients (Table 3, Quote 17), and felt that their organizations had no real desire to lead organizational change; wherein, DEI committees themselves become tokenized affairs (Table 3, Quote 18).

Subtheme 2b: Racialized RWCs’ perceptions of inclusion are affected by inherently racist/sexist societal norms and organizational practices governing the work environment

Participants shared the additional burdens they faced as a result of unstated workplace policies and culture on their racialized identity. Participants felt the ramifications of these unstated policies and culture through differential levels of support, unfavourable clinical standards, roadblocks in career advancement and overtly racist hiring practices (Table 3, Quotes, 19–22).

Participants also identified how organizational policies were perfunctorily implemented to ‘enhance’ workplace racial diversity; this was found to simply maintain the status quo. For example, a Black registered nurse perceived the hiring of racialized members as merely a tokenistic act of promoting pseudo-diversity within the workplace setting:

*“Trying to make it look like we’re okay*. *Like yeah*, *we do all of these other things but just to shut them up; let’s give them this one [bringing racialized person onto team]*. *That’ll shut them up for a little bit*. *‘Look they can’t say this because we have one here*.*”*

[RN7]

The maintenance of the racial status quo was also reflected through a lack of allyship. For example, a dietitian reported that working groups consisting solely of racialized individuals further contributed to the stagnation of transformational change within her workplace (Table 3, Quote 23). In contrast, a nurse reported the presence of white allies as a facilitator to advocating for BIPOC advancement up the career ladder (Table 3, Quote 24).

The *normalization* of whiteness lends itself to the *invisibilization or cloaking* of whiteness in the workplace setting. It was revealed that discussions of race rarely included discussions of whiteness or how whiteness operates to construct a racialized “Other”. Instead, as a result of the normalization of whiteness, RWCs were routinely questioned about their origins, their belonging, or their place in institutions. On the other hand, the normalization of whiteness means whiteness is never questioned, and implicitly is linked to authority and entitlement, which manifests in multiple ways (Table 3, Quote 25).

#### Theme 3: Differential treatment of RWCs had both positive and negative impacts on the participants’ relational, workplace and self- identity

Subtheme 3a: Having an intersectional identity as an RWC facilitated the provision of more comprehensive care to a diverse demographic of patients, while helping those patients more efficiently.

Participants reported being able to provide compassionate care stemming from a unique perspective drawing on their identity, culture, and experiences:

*“I’m more empathetic when it comes to the racialized population and understanding their struggle*, *where they might experience racism or where they might experience disenfranchisement*, *I just have that awareness*. *And I incorporate that into my practice*.*”*

[SW1]

Participants felt that an understanding of cultural and social nuances facilitated both rapport-building and lifestyle counseling, and that particularly with diabetes, establishing meaningful rapport was crucial in the provision of care. RWCs draw on their own experiences to meaningfully establish this through perspective and sensitivity:

*“I think rapport-building would probably be the biggest [factor] around culture*. *[…]Understanding that there’s a cultural sensitivity around the way in which self-management is in any chronic disease but definitely diabetes*, *and just appreciating that there are cultural differences and being sensitive to that*. *I think being racialized myself is actually a benefit to working with racialized patients*.*”*

[RN4]

Comprehending the unique obstacles presented to racialized patients, particularly racialized women, allowed RWCs to collaborate with their patients in finding realistic, tailored treatment regimens:

*“I’m more willing to recognize the impact of people’s culture*, *like people aren’t just going to give up rice and it’s not something to be angry about*. *It’s about figuring out how to talk to people about what they really value and shifting and supporting them to gain self-confidence and self-efficacy to change behavior*.*”*

[MD8]

Subtheme 3b: This intersectional identity resulted in deprofessionalization and an increased workload in an environment that was tailored to hinder the success of RWCs.

Participants asserted that they bore additional responsibilities, as well as pressure to overachieve beyond what is deemed necessary for one’s role, in comparison to their male and non-racialized counterparts. Participants identified broader societal norms of racism and gendered expectations as the source of increased workplace expectations they experienced. Some participants see these added meritocratic expectations as another obstacles; as one East Asian endocrinologist recounts:

*“My parents echoed that to say*, *to get to the same place*, *you were going to need to work harder*, *likely because of what general Canadians would view us but I don’t think it’s been a negative thing for me*. *I think I’ve just recognized I’m just gonna have to work hard*.*”*

[MD8]

One Black nurse practitioner commented on this systemic inequality in workplace expectations, elucidating how high the bar is for recognition and acceptance for less privileged demographics:

*“I always have this feeling that there’s a lot of room for improvement for the black person or the racialized person in this community*,*wherever this is*, *in Canada*, *in the workplace*. *I feel there’s a lot of work […]that you have to [do]*, *for you to be seen to be good*, *to be capable of doing what you’re doing and accepted*. *You have to go an extra mile and I don’t think that’s fair*.*”*

[NP1]

One Black nurse expressed that she considered resigning from her employment as discriminatory experiences accumulated, despite the risk of financial insecurity.

Subtheme 3c: This increased workload inevitably took its toll through diminished perceptions of their own capabilities, deficits in mental health, and depleted self-worth.

Participants felt burdened by their role as sole advocates:

*“People kind of sometimes look to me with the rise of the anti-Asian issues that are happening right now and will ask me how I feel about it*. *It’s great that we feel safe to have these conversations but it’s more of a burden I feel*.*”*

[RD3]

This additional burden in combination with lack of support culminated in poor psychological well-being; a Black nurse recounted how overwhelmed she felt when her manager abandoned her at a time crucial to her career progression:

*“I felt absolutely defeated and felt[…]*, *This is the* ***straw that broke the camel’s back***. *[…]I really was at an emotional breaking point*.*”*

[RN7]

This emotional burden elicited by racial and gender-based prejudice and expectations included exhaustion, anxiety and fear, and moral injury:

*“Absolutely impacted my self-confidence and self-worth and made me feel like I always have to overperform or stay on top of everything*. *So it definitely made me much more anxious*, *because I felt like any area that they felt was lacking would be used against me*. *They would try to compromise my employment status*.*”*

[RN7]

Resultantly, a diminished sense of self-worth became a common theme amongst many participants:

*“I’m just a cog in the wheel*, *and not something that the organization sees value in as more than just a worker*.*”*

[RD1]

*“I think when an authority figure you butt heads with*, *there’s that temporary shake in the foundation of who you think you are*. *You could be completely sure of yourself*, *but when there’s an authoritative figure who challenges you*, *you definitely would have that trepidation*.*”*

[RD4]

Importantly, participants noted their own individual sense of their gender and racial identity were a source of pride:

“I *feel proud; it makes me feel like I’m diverse and different*. *I find it to be positive in my life*. *I find it makes me more interesting*. *I think that that’s how it helps me with my sense of self*.”

[SW1]

Though participants differed in their responses to discriminatory practices, all noted the potential or reality of the negative impact of these practices on their self-worth, confidence, and sense of ability.

#### Mapping of findings to SEM

**Fig 2:**
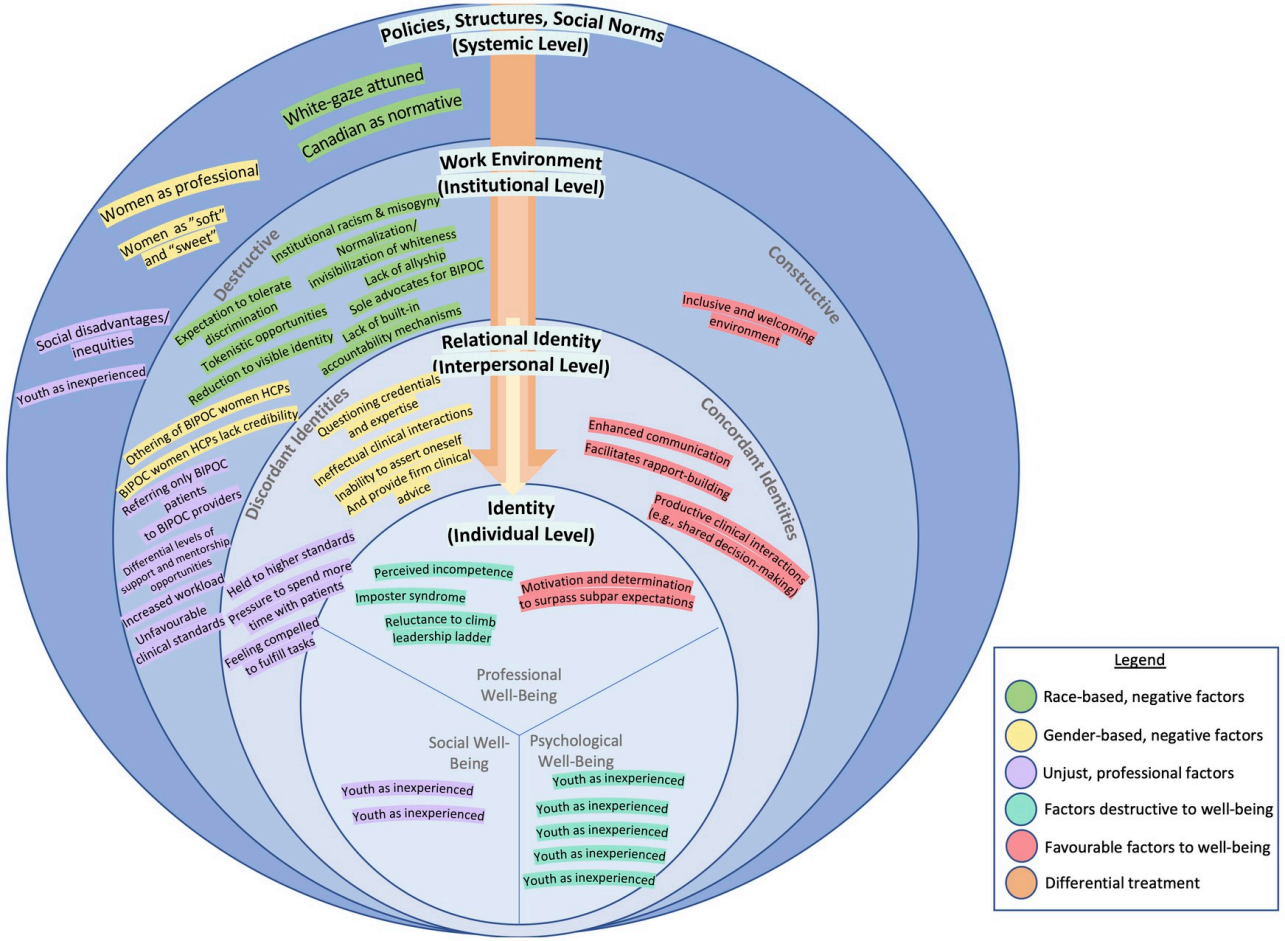
Selective codes from study themes, mapped to the adapted SEM constructs.

Codes from the themes identified above were mapped onto the SEM to explicate our findings and the interconnections between themes and sub-themes (Fig 2). A broader pattern of themes revealed themselves: differential treatment (Fig 2, orange arrows) of RWCs stems from far-reaching upstream policies, structures, and social norms (Fig 2, systemic/community level). These upstream policies, structures, and social norms then filter through different levels of the socio-ecological model, including work environments and communities (Fig 2, institutional level), which eventually impacts one’s relational identity (i.e., the identities co-constructed discursively through relationships) (Fig 2, interpersonal level), as well as one’s perception of oneself (Fig 2, individual level).

## Discussion

Using the SEM to identify linkages between constructs (i.e., Themes 1 and 2) and their impacts (i.e., Theme 3), our findings revealed two distinct ‘double-edged swords’ with respect to the participants’ racialized identity, and their role in representing BIPOC (Fig 2). Firstly, the participants’ racialized identities were observed to be advantageous for the provision of diabetes care, and were found to facilitate rapport (Fig 2, relational identity level). However, leveraging their intersectional identity to navigate their workplace interactions was also reported to cause undue psychological distress due to pressures to overachieve and overperform. Secondly, our findings demonstrated that the role of participants in representing BIPOC within their work environment was divided in its impact. Participants felt an inherent duty to act as advocates for their racialized patients and colleagues by taking on uncompensated roles on DEI committees. However, our findings revealed that these involvements were often tokenistic due to the reduction of these participants to merely their visible identity, without regard for their decision-making knowledge (Fig 2, work environment level). This consequent minority tax culminated in poor psychological well-being.

While relational rapport confers benefits to patients, the double-edged sword of paradoxical expectations exerts a substantial toll on the individual. For example, self-perceived incompetence contributes to imposter syndrome, first described by Clance in 1978 to describe high-achieving women, who upon completion of an achievement-related task, discount any positive feedback by attributing their success to effort or luck, which leads to self-doubt, depression, or anxiety [23]. One’s cumulative experiences of consistent, systematic othering, exclusion, and discrimination can lead to the development of imposter syndrome, a lack of belonging within the workplace, and feelings of proving oneself [23]. Participants cited impacts related to professional development, reluctance to “climb the leadership ladder”, and internalization of fear that they would come across as too aggressive if they attempted to establish authority in a clinical setting (Table 3, Quote 26).

To nurture professional development, there is a need for explicit conversations about career achievements and professional development between faculty members. This communication is essential in validation of one’s achievements, improving one’s confidence and reducing perceived incompetence. Faucett and colleagues (2021) detail how mentorship can facilitate these conversations by fostering the “psychological safety to define one’s ideal career” [24]. This includes learning how to express concerns against authority figures and learning to decline service requests when oversubscribed. Early career faculty can enjoy the psychological safety to take risks when supported by a mentor whose institutional authority provides a safety net.

Similarly, the “opportunity” to represent BIPOC is an arduous burden; this minority tax consists of disparities in five categories: responsibility for achieving diversity efforts, racism, isolation, lack of mentorship opportunities, and clinical and promotion-based inequities [25]. Our study identified examples of minority tax in each of these categories. The impact of this minority tax in academic medicine is reduced career advancement, and the relative absence of racialized clinicians in leadership [26]. These impacts have further downstream negative impacts, with less research in health care needs of racialized patients, and fewer mentors for racialized faculty [25]. “Tax reform” strategies suggested to reduce these impacts include “tax reduction” strategies such as removing the diversity-related committees and projects; “tax deferral” strategies such as engaging minority faculty when career promotion is underway; “standardizing tax” strategies such as encouraging white faculty to share responsibility for diversity initiatives; as well as “tax deduction” strategies such as giving appropriate credit (academic and financial) for diversity-related activities [27]. One method related to “tax deduction” is for promotions committees to formally recognize diversity-related initiatives by developing “committee activity points” (CAPs). These CAPs would be standardized and applied uniformly in promotion and compensation assessments for all faculty members. This strategy benefits both the institution and individual faculty members as the voices of minority faculty are heard, allowing the institution to benefit from minority representation, while equally ensuring that individual faculty members receive credit for these activities in promotion decisions [27]. Ensuring that clinical and community endeavors are counted toward promotion is essential in valuing diversity efforts fairly. Further, consistent tracking of diversity-related activities in monthly or quarterly reports creates a “well-defined footprint” for BIPOC promotion and advancement into leadership positions [27]. The development of CAPs may equally incentivize white faculty to engage in diversity-related endeavors. With reference to the “standardizing tax” initiative, this would promote white faculty to shoulder the responsibility for engaging in diversity work and lead to a change in the perception of diversity work, combating unconscious biases and discrimination. This would also provide additional time for BIPOC faculty to engage in leadership positions on non-diversity-related committees, widening their representation within the institution and power for decision-making [27].

The allostatic load of the ‘double-edged sword’ on participants’ well-being identified in our study also have negative implications to the provision of diabetes care. Ultimately, juggling paradoxical expectations leads to burnout, which is reported to impair the quality of care delivered to patients [28] and compromise patient safety [29]. Lower quality of care increases the risk of diabetes complications, developing comorbidities, and mortality [30]. This is of particular relevance to racialized patients, as provider burnout would further exacerbate the suboptimal quality of diabetes care they receive and pose barriers to self-management. It is therefore incumbent upon healthcare systems to address the unfairness of racism and prevent burnout among RWCs.

Structural racism embedded within the healthcare system is a primary contributor to race-based health inequities [31]. The normalization of whiteness and invisibilization of racial inequities as observed in our study affirms the need for greater cultural awareness and organizational practice of accountability. Diabetes is a chronic illness that requires team-based care [32, 33]; as such, all providers within a patient’s circle of care must be capable and adequately trained to tailor treatment plans to the patient’s specific capacities and capabilities [34]. However, to achieve this, the priority must be to alter the wider healthcare system that permits and perpetuates a culture of overt and covert discrimination, as recounted by the participants of this study.

Our study is limited by a few factors. First, there is limited data regarding the enablers of equity particularly at the macro levels, which is reflected in our SEM (i.e., limited labels for constructive factors). However, the absence of such enablers may be representative of the reality of the experiences of RWCs in diabetes care settings. Second, the data specific to diabetes care settings is also limited in our study. Again, this may also reflect the reality that the experiences of RWCs are not only experienced in one field, but rather may manifest similarly in different contexts of healthcare delivery. Furthermore, given the sensitive topics explored in this study, the mode of interviewing may have influenced the nature of the discussion as face-to-face interviewing often provides an added degree of safety and comfort during the interview that might not have been optimally achieved during the virtual interview for this study. Participant distress may have also been more difficult to identify and respond appropriately [35]. To mitigate some of these challenges, interviewers incorporated rapport-building techniques such as mutual attentiveness and common grounding behaviour from the start of the interview [36].

Key strengths of our study include the pan-Canadian context and diversity of participants by ethnicity and profession contributing to data triangulation of our findings; the various strategies employed to establish trustworthiness of our findings (e.g., researcher reflexivity, consensus-building) [37]; and the application of the SEM to comprehensively understand our findings within the context of the healthcare system.

## Conclusion

To achieve sustainable change, a multi-faceted strategy targeting individual, interpersonal, community, institutional and policy levels, is required [38]. For example, Hunter New England embarked on such a strategy, “Closing the Gap” in New South Wales, via mandatory staff training and education (e.g., Cultural Respect Education Program), leadership engagement and support (e.g., development of Counter-Racism Policy Compliance Procedure), and ongoing partnerships, negotiations and consultations (e.g., shared decision-making with Aboriginal parties) [39]. Strategies to optimize implementation include establishing leadership buy-in and commitment, investing dedicated resources and funding, engaging appropriate expertise, and establishing ongoing meaningful community and patient partnerships [38–40]. The differential treatment of RWCs arises predominantly from the macro systems of the work environment as well as policies, structures, and social norms. Accordingly, to address these disparities equitably, the burden needs to be shifted to the source–namely systems. Systemic interventions include valuing diversity efforts fairly (i.e., tax reform methods outlined above), instituting policies and procedures that address and correct bias (i.e., robust accountability systems for acts of discrimination, mandatory unconscious bias training), and prioritizing an inclusive and humanistic culture broadly across faculty and leadership (i.e., encourage positive curiosity when encountering “otherness”, explicit faculty retention strategies, transparent communications) [25, 40].

## Supporting information

S1 Table COREQ checklist criteria. (PDF)

S1 File Socioecological Model (SEM) framework, intersectionality, and theorizing gender and race [41–44]. (DOCX)

S2 File Positionality statements. (DOCX)
