# Supplementary material for: “The straw that broke the camel’s back”: An analysis of racialized women clinicians’ experiences providing diabetes care
Source: PLoS One. 2024 Jul 11;19(7):e0305473. doi: 10.1371/journal.pone.0305473 (PMC11239002; doi:10.1371/journal.pone.0305473)
Supplement: S1 File — (DOCX) [file pone.0305473.s002.docx]

**S2 File:** **Socioecological Model (SEM) Framework, Intersectionality, and theorizing gender and race**

We employed the socioecological model (SEM), a theoretical framework which posits that an individual’s health outcomes and experiences are impacted by, and occur within, a larger, dynamic network of intrapersonal relationships, relational factors, institutional processes, and societal norms and expectations [10]. The utility of the SEM is that it allows for analysis across all levels of influence, interaction, and norms. For example: we can readily analyze how individuals understand *their own* experiences of race and gender; we can also readily see how interpersonal interactions, institutional norms, and systemic/structural policies also shape individuals’ experiences of their racialized and gendered identities. The SEM does not necessarily clarify, in and of itself, what is meant by race, gender, or sex, though these also have implications for how we proceeded in our analysis.

In concert with considering the intersectionality of race, sex, and age, we used the SEM to understand the interaction between factors contributing to biased expectations of RWCs within diabetes care settings and the wider healthcare system. The paper also aims to draw on theories of intersectionality [12], which posit that experiences of neither gender nor race can be addressed *singularly*; rather, the *combined* experiential impact of race and gender affect racialized women at all levels of their professional lives. In Crenshaw’s seminal work, it was observed that Black women were discriminated against in a professional environment *despite* white women and Black men *not* experiencing the same levels of discrimination. Thus, the theory of intersectionality demands that we analyze the experiences of gender and race as occurring simultaneously.

It was with a view to discuss the *multiple levels* of racialized women clinicians’ professional lives, including individual (felt) racism/sexism, interpersonal interactions, institutional policies/procedures/norms, and broad-ranging systemic/structural factors. At *each* SEM level, intersectionality is present in how racialized women clinicians navigate experiences of misogyny, sexism, and racism.

In our conceptualization of gender and sex, we make a note here that sex and gender are not the same. When referencing sex here, we refer to sex assigned at birth. The authors acknowledge that individuals’ gender identities can vary (two of the authors are members of the queer community). Gender is learned, performed, and reified through actions occurring at all levels of the socioecological model; it is through a process of gendering whereby people acclimate, over their life-course to their gender identities. There is interplay between internal (felt) identity and externally mediated norms and expectations of gender or “sex” that people continually navigate. In this paper, we examine racialized women clinicians’ experiences of their racialized and gendered experiences at all levels of the SEM.

Furthermore, in our conceptualization, racism is not a static concept but a dynamic process that operates across systemic, structural, and individual levels [13]. When referring to "race" or "racialization," we view race as a socially and structurally constructed identity resulting from the process of racialization [41, 42]. This process can be traced back to European colonization and expansion, which led to land and resource theft [43] and the creation of the global of economic disparities between the global north and south, defined by economic disparities [44]. The effects of colonialism continue to shape the experiences of racialized individuals today. For the purposes of this paper, "racialized" refers to non-white identities. It is crucial to understand racialization as a historical and ongoing process stemming from colonial policies and practices, rather than as a fixed identity [13]. From a health outcomes perspective, race serves as an upstream determinant of health due to its influence on social and structural determinants.

Racism as a process can occur systemically through policies, practices, institutions. It can also occur socially through interpersonal interactions. These are often connected and correlated. Structural racism, systemic racism, and institutional racism, while distinct, are often interconnected. For the purpose of this paper, we observe racism at different levels of the social ecological model: for example, at the individual level, participants may hold certain beliefs or norms about their identities. At the interpersonal level, they may encounter other people’s ideas about their racial identity. At institutional and structural levels, racialized individuals navigate complex policies, regulations, and circumstances that exacerbate disparities in resources, and opportunities. Importantly, the processes of racialization due to colonialism are linked to Eurocentricity; as a result, differently racialized individuals experience different racial biases or stereotypes. For the purpose of this paper, we use the term "structural racism" to encompass all three forms of racism. Structural racism manifests through inequitable laws and regulations within various systems, including housing, education, employment, work-related benefits, credit, media, criminal justice, and healthcare. Importantly, racism is a process that can be conceptualized at all levels of the SEM, with mutually reinforcing elements. These systems mutually reinforce one another, perpetuating racial disparities and injustices.
