# Supplementary material for: “The straw that broke the camel’s back”: An analysis of racialized women clinicians’ experiences providing diabetes care
Source: PLoS One. 2024 Jul 11;19(7):e0305473. doi: 10.1371/journal.pone.0305473 (PMC11239002; doi:10.1371/journal.pone.0305473)
Supplement: S2 File — (DOCX) [file pone.0305473.s003.docx]

**S3 File: Positionality Statements**

AS is a first-year medical student with a Master’s degree in Public Health. She identifies as South-Asian and female.

SR is a first-year medical student. She identifies as South Asian and female.

DR is an intern physician. He identifies as an Egyptian and North American male.

TA is an endocrinologist engaged in transgender medicine and diabetes care in an urban quaternary care centre that provides care to diverse populations. She identifies as a queer South-Asian cisgender woman.

SH has a Master’s degree in Public Health and identifies as a South Asian queer cisgender woman.

CY is an endocrinologist engaged in diabetes care in an urban quaternary care centre that provides care to diverse populations; she identifies as South-East Asian and female.
